# Neighborhood deprivation and biomarkers of health in Britain: the mediating role of the physical environment

**DOI:** 10.1186/s12889-018-5667-3

**Published:** 2018-06-27

**Authors:** M. Pia Chaparro, Michaela Benzeval, Elizabeth Richardson, Richard Mitchell

**Affiliations:** 1 Institute for Social and Economic Research (ISER), University of Essex. Wivenhoe Park, Colchester, Essex, CO4 3SQ UK; 2 Present Address : Department of Global Community Health and Behavioral Sciences, School of Public Health and Tropical Medicine, Tulane University, 1440 Canal St., suite 2200-16, New Orleans, LA 70112 USA; 3 Institute for Health and Wellbeing, University of Glasgow, 1 Lilybank Gardens, Glasgow, G12 8RZ UK; 4 NHS Health Scotland, Gyle Square, 1 S Gyle Cres, Edinburgh, EH12 9EB UK; 5 Centre for Research on Environment, Society and Health (CRESH), School of GeoSciences, University of Edinburgh, Drummond Street, Edinburgh, EH8 9XP UK; 6 MRC/CSO Social and Public Health Sciences Unit, University of Glasgow, 200 Renfield Street, Glasgow, G2 3QB UK

**Keywords:** Socioeconomic deprivation, Biomarkers, Physical environment, Mediation analysis, Britain

## Abstract

**Background:**

Neighborhood deprivation has been consistently linked to poor individual health outcomes; however, studies exploring the mechanisms involved in this association are scarce. The objective of this study was to investigate whether objective measures of the physical environment mediate the association between neighborhood socioeconomic deprivation and biomarkers of health in Britain.

**Methods:**

We linked individual-level biomarker data from *Understanding Society: The UK Household Longitudinal Survey* (2010–2012) to neighborhood-level data from different governmental sources. Our outcome variables were forced expiratory volume in 1 s (FEV_1_%; *n*=16,347), systolic blood pressure (SBP; *n*=16,846), body mass index (BMI; *n*=19,417), and levels of C-reactive protein (CRP; *n*=11,825). Our measure of neighborhood socioeconomic deprivation was the Carstairs index, and the neighborhood-level mediators were levels of air pollutants (sulphur dioxide [SO_2_], particulate matter [PM_10_], nitrogen dioxide [NO_2_], and carbon monoxide [CO]), green space, and proximity to waste and industrial facilities. We fitted a multilevel mediation model following a multilevel structural equation framework in MPlus v7.4, adjusting for age, gender, and income.

**Results:**

Residents of poor neighborhoods and those exposed to higher pollution and less green space had worse health outcomes. However, only SO_2_ exposure significantly and partially mediated the association between neighborhood socioeconomic deprivation and SBP, BMI, and CRP.

**Conclusion:**

Reducing air pollution exposure and increasing access to green space may improve population health but may not decrease health inequalities in Britain.

**Electronic supplementary material:**

The online version of this article (10.1186/s12889-018-5667-3) contains supplementary material, which is available to authorized users.

## Background

The idea that our residential neighborhoods affect our health has been extensively studied [1–3]. A consistent finding is that residents of socioeconomically deprived neighborhoods have worse health outcomes than residents of non-deprived neighborhoods, even after adjusting for individual-level socioeconomic position (SEP) [1, 4, 5]. This suggests that certain neighborhood characteristics may affect health, for example, social and economic facilities, environmental hazards, climate, social capital, etc. [6, 7], or that the stress associated with living in poor neighborhoods directly affects health [1]. In order to design interventions to reduce socio-spatial health inequalities, it is important to understand the mechanisms by which neighborhood deprivation affects the health of individuals.

To date, while a number of studies have identified potential mechanisms linking neighborhood socioeconomic deprivation and health (e.g. [1, 2]), few have actually explored them empirically. For example, a recent systematic review of multilevel studies investigated the interactive and independent associations of neighborhood deprivation and objective measures of the built environment on individual-level health [8]. Of the 33 studies included, only one investigated potential mediators between neighborhood deprivation and health outcomes – specifically, the neighborhood availability of alcohol as a mediator between neighborhood deprivation and individual alcohol consumption [9]. The vast majority of the included studies assessed *moderation* effects, or how neighborhood deprivation interacted with other aspects of the environment to affect health [8]. The authors concluded that “there is still a lack of knowledge to what extent the built environment mediates effects of neighborhood SEP on individual health” [8].

Those studies which have explored mediation in relation to neighborhood socioeconomic deprivation and individual health focused mostly on self-reported *social* environmental characteristics [10–13]. For example, two studies based on a community survey in Detroit, Michigan, USA found that a combination of perceived social (e.g. disorder, crime) and physical (e.g. exposure to air pollution) neighborhood characteristics partly explained the association between neighborhood poverty and allostatic load. However, these characteristics were measured subjectively and at the individual level [11, 12].

Objective measures of the *physical* environment have seldom been assessed as possible mediators in the neighborhood deprivation – health relationship, despite their likely role in this association [1]. Exposure to air pollution, for example, has been linked to poor lung function [14, 15], increased blood pressure [15, 16], obesity development [17–19], and markers of inflammation [20, 21], and socioeconomically deprived neighborhoods are *on average* more polluted than those less deprived [22, 23]. Similarly, the positive effects of exposure to green space on health have been widely documented [24–26], and green space exposure is also socially patterned [27, 28].

Aspects of the physical environment can have an impact on health both through biological and psychosocial mechanisms [29]. Exposure to air pollution affects lung function via inflammation of the airways, primarily through the production of free radicals in the lungs [30]. Air pollution is hypothesized to increase blood pressure due to an increase in endothelin-1, which is produced in the lungs in response to the presence of free radicals and which is involved in maintaining vascular tone [31]. Pollution has also been linked to an increased obesity risk with a proposed inflammatory pathway triggering visceral fat deposition [18, 19], while inactivation of antioxidant enzymes has been proposed as a mechanism linking air pollution and increased systemic inflammation [32]. Exposure to green space has been hypothesized to impact health by facilitating social interactions [33]; promoting physical activity [34]), which in turn might reduce body mass index and blood pressure and improve (or not lead to significant declines in) lung function; and – most importantly – by directly reducing stress-response [34, 35], which often manifest itself via inflammation processes. Proximity to waste and industrial facilities could influence health by increased exposure to toxic emissions [36, 37] or via a psychosocial pathway: proximity to waste and industrial facilities has been linked to stress and decreased psychological well-being [38–40].

In addition, it is possible that exposure to certain neighborhood physical environments may affect the health of some sub-populations differently. For example, research shows that the elderly [30, 31] and smokers [41] may be more susceptible to the effects of air pollution, whereas women seem to be more affected by their neighborhood environments than men, likely because of an increased social susceptibility and/or exposure [42, 43]. Finally, residents of urban settings and larger cities like London may have different levels of exposure to certain environmental characteristics at similar levels of deprivation than those living in rural areas or smaller cities.

Given the plausible pathways, but lack of evidence testing them, the aim of this study was to investigate the contribution of some key physical neighborhood characteristics (air pollution, exposure to green space, and proximity to waste and industrial facilities) to social inequalities in health among the British population. We chose the outcomes of the study – lung function, blood pressure, body mass, and inflammation – based on existing evidence linking them to both neighborhood socioeconomic deprivation [44–46] and physical environment measures [14, 16, 19, 21], as noted above. We ran mediation analysis with all potential mediators simultaneously and hypothesized that: 1) individuals living in socioeconomically deprived neighborhoods would have a higher exposure to a poor physical environment (with higher pollution, lower availability of green space, closer proximity to waste/industrial facilities); 2) individuals living in socioeconomically deprived neighborhoods will have worse health outcomes; and 3) part of the association between neighborhood socioeconomic deprivation and health outcomes will be explained by a higher exposure to a poor physical environment.

## Methods

### Individual-level data and outcome variables

Individual-level data came from waves 2 and 3 of *Understanding Society: The UK Household Longitudinal Survey* (UKHLS) [47–49]. UKHLS is a longitudinal study of ~ 40,000 households at wave 1 designed to be representative of all countries in the United Kingdom [50]. Data collection started in 2009 with households selected following a multi-stage clustered sample design [50]. In wave 2, the British Household Panel Study (BHPS) was incorporated into Understanding Society. In waves 2 (UKHLS sample) and 3 (BHPS sample) (2010–2012), a nurse visit was conducted that included direct health assessments and blood measurements (i.e. biomarkers) [51]. The eligibility criteria for participation included completion of the main interview survey; being ≥16 years; completion of the interview in English; and, for women, not being pregnant [51]. Only participants living in England, Wales, and Scotland were included. The nurse assessment was undertaken by the National Centre for Social Research, who trained registered nurses on data collection and study protocols [51]. Since we are using UKHLS data from waves 2 and 3 only (combined), this study followed a cross-sectional design.

Details related to the recruitment protocol for the nurse visit can be found elsewhere [51]. Briefly, participants fulfilling the eligibility criteria were visited by a nurse ~ 5 months after the main interview for collection of blood sample and health measures. Participants were allowed to decline any given procedure or measurement at any time, and were given a £10 voucher upon completion of the nurse visit. Approval from the National Research Ethics Service was obtained for data collection (Oxfordshire A REC, Reference: 10/H0604/2).

From an eligible sample of 35,875 participants, 20,644 completed the nurse health assessment (57.5% response rate) and 13,107 provided blood and had at least one biomarker extracted (37% response rate) [52]. We used data from both the health assessment and the blood test samples, with different sample sizes for each of the outcomes under study, as explained below.

The outcome variables included were lung function, blood pressure, body mass index (BMI), and levels of C-reactive protein (CRP). Lung function was assessed with two different spirometers measuring peak expiratory flow: in England and Wales with the NDD Easy On-PC spirometer, and in Scotland with the Vitalograph Escort [51]. Given that the equivalence of these two machines cannot be ascertained, we only included lung function measures for England and Wales.

Forced expiratory volume in 1 s (FEV_1_), the amount of air that can be blown out in one second, was used as a measure of lung function. Percent predicted FEV_1_ (FEV_1_%) was estimated from the average of three valid readings following the European Respiratory Society Global Lung Function Initiative (ERS-GLI) equations, adjusting for age, gender, height and ethnicity [53]. Implausible FEV_1_% values (< 20% or > 200%) were eliminated (*N* = 8) for a total analytical sample of 16,347 participants.

Blood pressure was measured with the portable monitor Omron HEM 907. Three measurements were taken on the right arm, with the participant sitting down [50]; the mean of valid readings was used. For this study, we focused on systolic blood pressure (SBP), which was adjusted for participants taking blood pressure medication (21.8% of the sample) by adding + 10 mmHg to their readings [54]. The sample size of participants with valid SBP readings was 16,846.

BMI (kg/m^2^) was estimated from measured weight and height. Height was measured with a portable stadiometer, with the participant wearing no shoes [51]. Weight was measured with a Tanita BF 522 digital floor scale with the participant wearing no socks or shoes. Only one measurement to the nearest millimeter and 0.1 kg, respectively, was taken. Participants who weighed > 130 kg were asked to estimate their weight as the scales used are inaccurate above this level (*N* = 163) [51]. Only participants who were ≥ 18 years were included in models with BMI as BMI for people < 18 years (*N* = 438) can only be interpreted in reference to age- and sex-specific growth curves. Implausible BMI values (< 15 or > 60) were eliminated (*N* = 17) for a total analytical sample of 19,417 participants.

CRP, an inflammatory marker, was obtained from non-fasting blood samples and analyzed from serum using the N Latex CRP mono immunoassay on the Behring Nephelometer II Analyzer (Dade Behring, Milton Keynes, UK; *N* = 12,530) [52]. CRP values > 10 mg/L most likely reflect a current infection instead of chronic inflammation; therefore, 705 participants with such CRP values were excluded for a final analytical sample of 11,825.

### Neighborhood-level data and exposure and mediator variables

Neighborhood was defined by assigning the residential location of the participant at the time of data collection to the 2001 Census Area Statistic (CAS) ward (*N* = 4929 CAS wards; average 4.2 individual per CAS ward). CAS ward is a small areal unit (mean population 5518) used in the collection of census data [55]. It has been widely used in studies of physical environment and health (e.g. [56, 57]). Neighborhood-level data included our exposure variable, the Carstairs index, as a measure of neighborhood socioeconomic deprivation [58], and individual components of the Multiple Environmental Deprivation Index (MEDIx) [56] as physical environment measures (mediators). MEDIx is only available at the CAS ward level and all other area-level variables used in this study were also available for CAS wards. Although not ideal, CAS wards were deemed acceptable for this analysis as they are small enough to be sensitive to finer scale environmental variations, large enough to be robust in ecological analyses [56], and remain the unit which has been most commonly used for this kind of work in the UK.

The Carstairs index was obtained from the UK 2001 Census [59]. This index was chosen as it is available across all three countries of Great Britain, and for the same time period as the physical environment data. The index is composed of four unweighted variables at the neighborhood-level: 1) unemployment, defined as the proportion of unemployed males ≥16 years; 2) overcrowding, defined as the proportion of households having more than one person per room; 3) car ownership, defined as the proportion of households with no car; and 4) low social class, defined as the proportion of households in which the head of the household belongs to social class IV or V [58]. In the 2001 Census, the measure of social class is based on the National Statistics Socioeconomic Classification (NS-SEC), which did not have information on social classes IV and V per se; as explained in detail elsewhere [60], an approximation of these classes was used instead. Each of these four variables is converted into a z-score (i.e. standardized) before the index is created. The Carstairs index, then, is the sum of the z-scores for each of these four variables, with higher values indicating greater socioeconomic deprivation.

MEDIx is a multivariate area-level indicator, inspired by indices like Carstairs, but reflecting physical environmental conditions rather than socioeconomic. MEDIx components were obtained from a variety of sources including the National Atmospheric Emissions Inventory, the European Pollutant Emission Register, the Generalised Land Use Database, and Coordination of Information on the Environment land cover data [56]. In this study, MEDIx was not used as an index since we hypothesized that some of its components may influence different biomarkers in different ways. The measures used were: 1) air pollution, including population weighted means at the CAS-level of sulphur dioxide (SO_2_; μg/m^3^; mean of annual averages 1999–2003), particulate matter (PM_10_; μg/m^3^; 1999–2003), nitrogen dioxide (NO_2_; μg/m^3^; 1999–2003), and carbon monoxide (CO; mg/m^3^; 2001–2006); 2) exposure to green space, defined as the percent of (public) green space in the CAS ward of residence (2000–2001); and 3) proximity to waste or industrial facilities, defined as the proportion of the CAS ward population living within 4 Km of a waste site or 1.6 Km from a metal processing/production site (2001–2002).

#### Covariates

Household income (operationalized as the log of monthly net household equivalised income, in pounds) was added in all models as a potential confounder since it may influence where people live and therefore the level of neighborhood socioeconomic deprivation a person is exposed to, as well as respondents’ biomarker levels. Missing income values were imputed as described elsewhere [50]. In models with SBP, BMI, and CRP as outcomes, age (years) and gender were added as covariates. Age and gender were not included in the FEV_1_% model because they were already accounted for in the estimation of FEV_1_%. We were unable to include an indicator of respondents’ ethnicity into our analysis since the UKHLS nurse sub-sample is primarily White/European (97%).

### Statistical analysis

#### Descriptive and bivariate analyses

Descriptive statistics were used to characterize the sample. Linear regression models were used to assess the bivariate associations between Carstairs index (exposure) and the physical environment measures (mediators) to assess if the associations were in the expected direction. We then tested associations between Carstairs and biomarkers (outcomes), and between physical environment measures and biomarkers, via multilevel linear regression models. These models were adjusted by household income (all outcomes), age and gender (all outcomes but FEV_1_%). Descriptive statistics, simple and multilevel linear regression models were carried out in SAS v9.4 (SAS Institute Inc., Cary, NC, USA).

#### 2–2-1 Multilevel mediation models

**Fig. 1:**
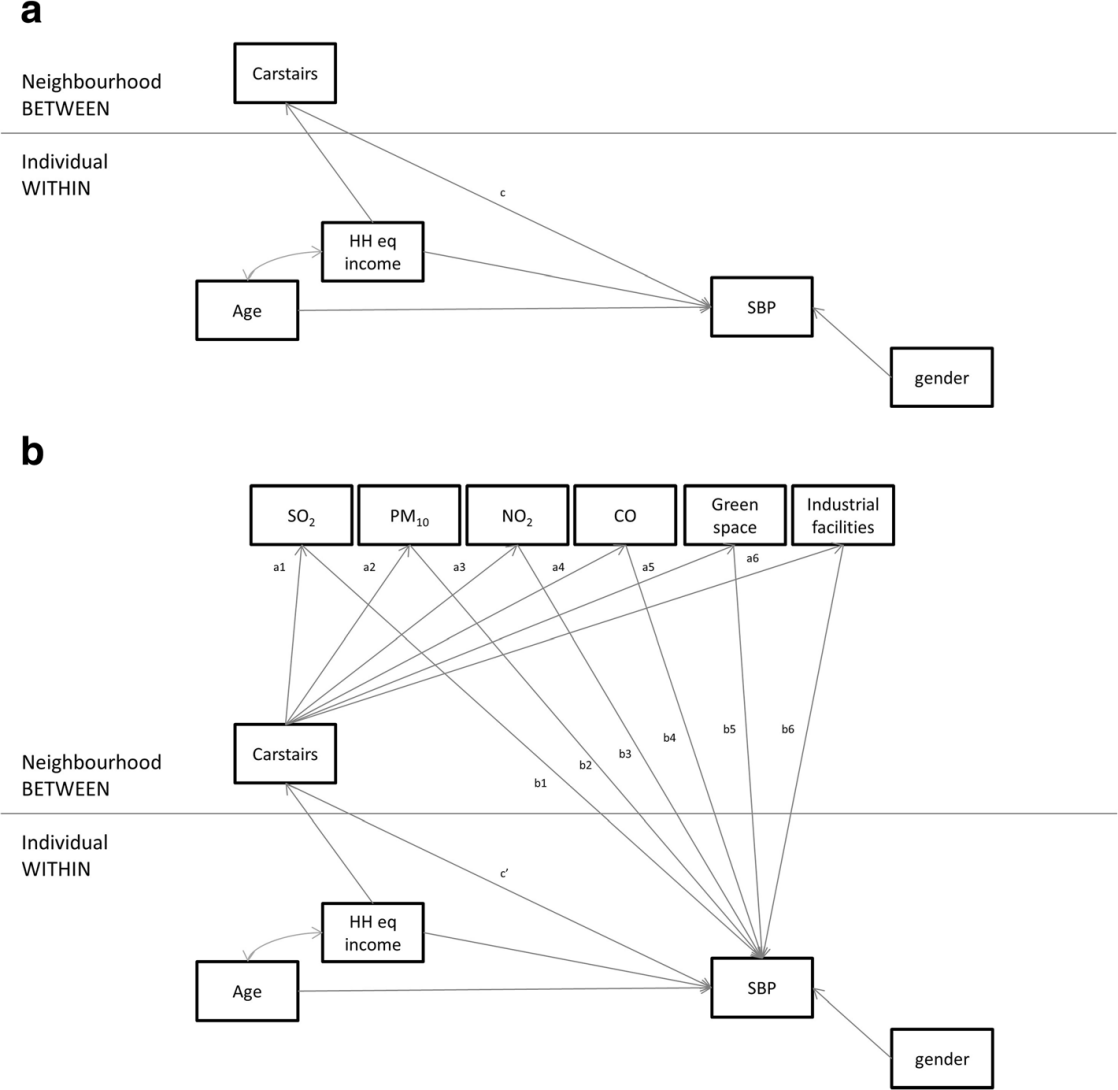
Analytical framework for the 2–2-1 multilevel mediation model proposed, using systolic blood pressure (SBP) as an outcome. **a**: Total effects of Carstairs (exposure) on SBP (outcome) without the inclusion of the mediators (path c). **b**: Direct effects of Carstairs on SBP, net of physical environment (mediators, paths c’, a_1_-a_7_, and b_1_-b_7_)

Given that: i) data were composed of individuals (level 1) nested within neighborhoods (level 2); ii) both the exposure (Carstairs) and the mediators (physical environment measures) were at level 2; iii) the outcome variables (biomarkers) were at level 1; and iv) we had multiple mediator variables, we fitted a 2–2-1 multilevel mediation model with multiple mediators in Mplus v7.4 following a multilevel structural equation modeling (MSEM) framework [61], as described by Preacher et al. [62, 63]. The operationalization of this multilevel mediation analysis is shown in Fig. 1. The coefficient *c* in Fig. 1a provided information on the total effect of Carstairs on SBP (as an example), whereas the coefficient *c’* in Fig. 1b provided the direct effect of Carstairs on SBP, net of the physical environment. The indirect or mediated effects were then estimated by assessing the association between Carstairs and the physical environment measures (*a*_*x*_ coefficients) and the association between the physical environment measures and SBP (*b*_*x*_ coefficients), by calculating *a*_x_*b_*x*_.

The MSEM framework allowed us to fit the model depicted in Fig. 1b in one step rather than fitting the 2–2 (exposure – mediators) and the 2–1 (mediators – exposure) components separately and computing the indirect effects manually (e.g. [64]). In addition, the MSEM framework allowed us to assess the impact of all mediators at the same time, while accounting for the correlation of their error terms. However, one disadvantage of this approach is that it does not handle categorical variables, therefore limiting our ability to seek ‘threshold’ effects by categorizing variables. Separate models were fitted for each of the outcome variables using maximum likelihood with robust standard errors (MLR) estimators. All models were unweighted given that multilevel weights were not available (CAS wards were not part of the sampling design of UKHLS) [65]. Therefore, our results may not be representative of residents of Great Britain.

#### Moderation effects

We investigated whether moderation effects existed by age (< 35 years, 35–60, and > 60 years) and gender. We also investigated moderation by smoking status (never, former, and current smoker) since smokers may be more susceptible to the effects of air pollution [41]. To account for variation in environmental setting which may have affected both the absolute levels of environmental characteristics and levels of exposure, we assessed moderation by urbanity (urban vs. rural), London residency, by country (England, Wales, and Scotland), and by ACORN classification. We only report on the results by urbanity and London residency here, to contain the size of the paper and because the analyses by country and ACORN did not help illuminate the interpretation of the results any further. Given the complexity of the 2–2-1 multilevel mediation models, we investigated moderation by stratified models, rather than by interaction terms.

#### Sensitivity analysis

We had access to a subset of data on some of the physical environment measures (air pollutants SO_2_, PM_10_, and NO_2_ only) at a finer geographical scale – Lower Level Super Output Areas (LSOAs) – and also for 2011, which is closer to the date in which the biomarker data was collected (2010–2012). We ran similar analyses to the ones described above with these pollutants as mediators, Carstairs 2011 as the predictor, and all biomarkers as outcomes. The results of this sensitivity analysis are not qualitatively different to those of the full models (as displayed in Fig. 1). Therefore, we decided to only present the results of the full models with all physical environment measures (all air pollutants, green space, and proximity to waste/industrial facilities) as mediators. Results of this sensitivity analysis, however, are available from the authors upon request.

## Results

Overall, the sample was composed of relatively healthy individuals in terms of FEV_1_%, SBP, and CRP (mean values within normal ranges), but who were on average overweight (mean BMI = 28 kg/m^2^; Table 1). Approximately 56% of the sample was female and the mean age was 51 years.

**Table 1:** Characteristics of the sample

	N	Mean	SE	Range
Individual-level variables				
Age (years)^a^	20,573	50.71	0.13	16,102
Household equivalised income (monthly, £)^a^	20,559	1726.22	17.34	− 3748.61, 130,362.60
Forced expiratory volume in 1 s (FEV_1_%)	16,347	92.46	0.13	22.07, 191.51
Systolic blood pressure (SBP, mmHg)	16,846	128.13	0.14	75.00, 226.00
Body Mass Index (BMI, kg/m^2^)	19,417	28.04	0.04	15.24, 59.64
C-reactive protein (CRP, mg/L)	11,825	2.08	0.02	0.20, 10.00
Neighborhood-level variables				
Carstairs index^b^	4929	0.16	3.26	−5.07, 15.27
Sulphur dioxide (SO_2_, μg/m^3^)^c^	4929	4.26	2.16	0.52, 19.14
Particulate matter (PM_10_, μg/m^3^)^c^	4929	15.63	2.05	9.30, 22.54
Nitrogen dioxide (NO_2_, mg/m^3^)^c^	4929	24.32	8.62	1.52, 60.36
Carbon monoxide (CO, μg/m^3^)^c^	4929	0.22	0.06	0.13, 0.56
%Green space	4929	57.28	27.51	0.00, 95.19
Proximity to industrial facilities^d^	4929	0.06	0.19	0.00, 1.00

^a^Age and household income values are based on the largest possible sample size

^b^Based on the following neighborhood characteristics: male unemployment, overcrowding, car ownership, and social class

^c^Population weighted, annual averages

^d^Proportion of neighborhood residents living within 4 Km of a waste site and/or 1.6 Km from a metal processing/production site

In bivariate analysis, higher Carstairs scores (i.e. higher socioeconomic deprivation) were significantly associated with higher levels of pollutants, lower proportion of green space, and closer proximity to waste/industrial facilities (e.g. a one unit increase in the Carstairs score was associated with 0.33 µg/m3 less SO2 in the neighborhood; Table 2). As shown in Table 3, neighborhood deprivation was associated with biomarkers in the expected direction: negatively with FEV_1_% (a one unit increase in the Carstairs index was associated with a decrease in FEV_1_% of 0.56),, and positively with SBP, BMI, and CRP (a one unit increase in Carstairs was associated with 0.18 mmHg higher SBP, 0.16 kg/m^2^ higher BMI, and 0.06 mg/L higher CRP). In terms of the individual association between physical environment variables and biomarkers, the results were mixed. Most associations were in the expected direction (e.g., higher levels of neighborhood SO_2_ linked to lower FEV_1_% and higher SBP, BMI and CRP), but some associations were in an unexpected direction (e.g., higher levels of neighborhood PM_10_, NO_2_, and CO linked to *lower* SBP), or not statistically significant (e.g. green space and BMI; Table 3).

**Table 2:** Bivariate associations between Carstairs index and physical environment variables (*N* = 4929 neighborhoods)

	Carstairs index		
	Estimate	SE	95%CI
Sulphur dioxide (SO_2_, μg/m^3^)^a^	0.33	0.02	0.29, 0.37
Particulate matter (PM_10_, μg/m^3^)^a^	0.32	0.02	0.28, 0.36
Nitrogen dioxide (NO_2_, mg/m^3^)^a^	0.11	0.01	0.10, 0.12
Carbon monoxide (CO, μg/m^3^)^a^	24.51	0.65	23.22, 25.79
%Green space	−0.055	0.001	−0.058, − 0.052
Proximity to industrial facilities^b^	1.01	0.24	0.54, 1.48

^a^Population weighted, annual averages

^b^Proportion of neighborhood residents living within 4 Km of a waste site and/or 1.6 Km from a metal processing/production site

**Table 3:** Associations between Carstairs index and biomarkers, and between physical environment variables and biomarkers (multilevel linear regression models)^a^

	FEV_1_% B (95% CI)	SBP (mmHg) B (95% CI)	BMI (kg/m^2^) B (95% CI)	CRP (mg/L) B (95% CI)
Carstairs index^b^	**− 0.563 (− 0.650, − 0.475)**	**0.181 (0.096, 0.266)**	**0.157 (0.129, 0.184)**	**0.056 (0.044, 0.068)**
Sulphur dioxide (SO_2_, μg/m^3^)	**−0.173 (− 0.295, − 0.050)**	**0.251 (0.133, 0.369)**	**0.105 (0.066, 0.144)**	**0.032 (0.015, 0.049)**
Particulate matter (PM_10_, μg/m^3^)	**−0.509 (− 0.665, − 0.353)**	**−0.364 (− 0.497, − 0.232)**	−0.039 (− 0.084, 0.005)	0.019 (− 0.000, 0.038)
Nitrogen dioxide (NO_2_, mg/m^3^)	**−0.122 (− 0.157, − 0.087)**	**−0.064 (− 0.095, − 0.032)**	−0.004 (− 0.015, 0.007)	0.004 (− 0.000, 0.008)
Carbon monoxide (CO, μg/m^3^)	**−19.566 (− 23.975, − 15.156)**	**−5.800 (− 10.091, − 1.509)**	0.196 (−1.242, 1.634)	**1.199 (0.574, 1.823)**
%Green space	**0.042 (0.032, 0.053)**	0.009 (− 0.000, 0.019)	−0.002 (− 0.006, 0.001)	**−0.003 (− 0.004, − 0.001)**
Proximity to industrial facilities	**−1.461 (− 2.824, − 0.097)**	0.800 (− 0.496, 2.097)	**0.681 (0.246, 1.117)**	**0.257 (0.070, 0.444)**

*FEV*_*1*_*%* percent predicted forced expiratory volume in 1 s, *SBP* systolic blood pressure, *BMI* body mass index, *CRP* C-reactive protein

^a^Model for FEV1% was adjusted by the log of household equivalised income. Models with the other outcomes were adjusted by age, gender, and the log of household equivalised income

^b^Higher value indicates higher deprivation

Bolded data are significant

The results of the multilevel mediation models for each outcome are shown in Table 4, which should be read in conjunction with Fig. 1b for ease in interpretation. The *total effects* of neighborhood socioeconomic deprivation on biomarker levels followed the same pattern and were of similar magnitude as those observed in Table 3 from simpler multilevel models (Table 4, first row, *c* pathway vs. first line of Table 3). Looking at the *a*_*1*_*-a*_*6*_ pathways (Fig. 1b and Table 4) we observe that the Carstairs index is associated with all physical environment variables in the expected direction. In turn, only some of the physical environment variables are associated with the biomarkers in the presence of Carstairs and with all mediators combined in one model (*b*_*1*_*-b*_*6*_ pathways, Table 4). Neighborhood exposure to SO_2_ was associated with all biomarkers except for FEV_1_%: those exposed to a higher level of SO_2_ in the neighborhood had on average a higher SBP, BMI, and CRP. In addition, proximity to waste/industrial facilities was associated with a higher BMI (*b*_*6*_ pathway for BMI) and a higher exposure to PM_10_ was associated with a higher CRP (*b*_*2*_ pathway for CRP). As above, levels of PM_10_, CO, and NO_2_ in the neighborhood were negatively associated with SBP, BMI, and CRP respectively (i.e. higher levels of these pollutants were associated with better health, net of Carstairs and all other physical environment variables).

**Table 4:** Results of the multilevel mediation models between Carstairs (exposure), physical environment variables (mediators), and biomarkers (outcomes)

	FEV_1_% (England and Wales only)^a^		SBP^b^		BMI^c^		CRP^d^	
	Estimate	95% CI	Estimate	95% CI	Estimate	95% CI	Estimate	95% CI
Total effects (Carstairs predicting biomarker, no mediators, c path)								
Carstairs (*c*)	**−0.617**	**− 0.706, − 0.528**	**0.191**	**0.105, 0.277**	**0.154**	**0.124, 0.183**	**0.060**	**0.048, 0.073**
Direct effects (Carstairs predicting biomarker net of mediators; c’, a_1_-a_7_, and b_1_-b_7_ paths)								
Within								
Age	–		**0.468**	**0.455, 0.482**	**0.044**	**0.039, 0.049**	**0.015**	**0.013, 0.018**
Gender	–		**−6.859**	**−7.315, −6.403**	−0.098	−0.238, 0.043	**0.292**	**0.221, 0.362**
Log income	**3.990**	**2.716, 5.264**	−1.024	−2.090, 0.041	0.124	−0.230, 0.478	**−0.298**	**− 0.454, − 0.142**
Between								
Carstairs (*c’*)	**−0.548**	**− 0.653, − 0.442**	**0.212**	**0.112, 0.312**	**0.182**	**0.148, 0.215**	**0.056**	**0.041, 0.071**
Log income (*on Carstairs*)	**−9.143**	**−10.420, −7.865**	**− 9.772**	**− 11.744, − 7.801**	**−9.761**	**−11.727, − 7.795**	**−9.777**	**−11.752, − 7.801**
Carstairs predicting mediators:								
SO_2_ (*a*_*1*_)	**0.181**	**0.161, 0.201**	**0.147**	**0.129, 0.165**	**0.147**	**0.129, 0.165**	**0.147**	**0.129, 0.165**
PM_10_ (*a*_*2*_)	**0.186**	**0.169, 0.203**	**0.127**	**0.108, 0.146**	**0.127**	**0.108, 0.146**	**0.127**	**0.108, 0.146**
NO_2_ (*a*_*3*_)	**0.940**	**0.867, 1.012**	**0.791**	**0.717, 0.864**	**0.791**	**0.717, 0.864**	**0.791**	**0.717, 0.864**
CO (*a*_*4*_)	**0.010**	**0.010, 0.011**	**0.009**	**0.009, 0.010**	**0.009**	**0.009, 0.010**	**0.009**	**0.009, 0.010**
Green space (*a*_*5*_)	**−4.184**	**−4.381, −3.986**	**−3.911**	**−4.097, − 3.724**	**−3.911**	**−4.097, − 3.724**	**−3.911**	**−4.097, − 3.724**
Industrial facilities (*a*_*6*_)	**0.004**	**0.002, 0.006**	**0.004**	**0.002, 0.005**	**0.004**	**0.002, 0.005**	**0.004**	**0.002, 0.005**
Mediators predicting biomarker:								
SO_2_ (*b*_*1*_)	0.066	−0.075, 0.208	**0.424**	**0.285, 0.562**	**0.098**	**0.056, 0.140**	**0.023**	**0.003, 0.043**
PM_10_ (*b*_*2*_)	−0.134	−0.491, 0.223	**− 0.661**	**−0.961, − 0.361**	−0.093	− 0.195, 0.009	**0.051**	**0.005, 0.097**
NO_2_ (*b*_*3*_)	−0.012	−0.121, 0.097	0.002	−0.098, 0.101	0.016	−0.018, 0.050	**− 0.023**	**−0.039, − 0.008**
CO (*b*_*4*_)	0.376	−11.969, 12.721	2.783	−7.933, 13.499	**−5.089**	**−8.907, −1.270**	1.196	−0.510, 2.901
Green space (*b*_*5*_)	0.011	−0.005, 0.027	0.007	−0.007, 0.021	0.000	−0.005, 0.004	0.000	−0.002, 0.002
Industrial facilities (*b*_*6*_)	−0.846	−2.199, 0.506	0.707	−0.613, 2.028	**0.554**	**0.117, 0.991**	0.173	−0.026, 0.371
Indirect effects (a*b)								
Between								
Total Indirect	**−0.069**	**−0.132, − 0.007**	−0.021	− 0.077, 0.034	**−0.028**	**− 0.047, − 0.009**	0.004	−0.004, 0.012
SO_2_ (*a*_1_*b_*1*_)	0.012	−0.014, 0.038	**0.062**	**0.041, 0.084**	**0.014**	**0.008, 0.021**	**0.003**	**0.000, 0.006**
PM_10_ (*a*_2_*b_*2*_)	−0.025	−0.091, 0.042	**− 0.084**	**−0.124, − 0.044**	−0.012	− 0.025, 0.001	**0.006**	**0.001, 0.012**
NO_2_ (*a*_3_*b_*3*_)	−0.011	−0.114, 0.091	0.001	−0.077, 0.080	0.013	−0.014, 0.039	**− 0.018**	**−0.031, − 0.006**
CO (*a*_4_*b_*4*_)	0.004	−0.123, 0.131	0.025	−0.072, 0.122	**− 0.046**	**−0.081, − 0.011**	0.011	−0.005, 0.026
Green space (*a*_5_*b_*5*_)	−0.045	−0.112, 0.022	− 0.029	−0.084, 0.026	0.001	−0.017, 0.019	0.001	−0.007, 0.009
Industrial facilities (*a*_6_*b_*6*_)	−0.004	−0.010, 0.002	0.002	−0.002, 0.007	**0.002**	**0.000, 0.004**	0.001	0.000, 0.001
Model fit statistics	X^2^ = 178.847, df = 12, p < .0001; RMSEA = 0.027; CFI = 0.991; TLI = 0.972, SRMR W = 0.003; SRMR B = 0.049		X^2^ = 258.072, df = 14, *p* < .0001; RMSEA = 0.029; CFI = 0.990; TLI = 0.971, SRMR W = 0.018; SRMR B = 0.046		X^2^ = 264.759, df = 14, p < .0001; RMSEA = 0.029; CFI = 0.988; TLI = 0.965, SRMR W = 0.018; SRMR B = 0.050		X^2^ = 263.910, df = 14, p < .0001; RMSEA = 0.029; CFI = 0.988; TLI = 0.964, SRMR W = 0.017; SRMR B = 0.047	

*FEV*_*1*_*%* percent predicted forced expiratory volume in 1 s, *SBP* systolic blood pressure, *BMI* body mass index, *CRP* C-reactive protein, Bolded data are significant

The indirect or mediated effects are displayed in the last panel of Table 4 (a*b). We observed no mediation effects for FEV_1_% for the individual physical environment measures, but there was a significant overall mediation effect (Table 4, total indirect). The total effect of Carstairs on FEV_1_% was − 0.617 (pathway *c*), meaning, a one-unit increase in the Carstairs index was associated with a decrease in FEV_1_% of 0.617. Even though each individual physical environment measure did not mediate the association between Carstairs and FEV_1_%, the association between Carstairs and FEV_1_% was reduced to − 0.548 (pathway *c'*) because of the presence of the physical environment measures in the model (total indirect effect = -0.069). In turn, neighborhood SO_2_ partly mediated the association between Carstairs and SBP, BMI, and CRP (see Table 4, *a*_1_*b_*1*_ effects): neighborhoods with greater socioeconomic deprivation had higher levels of SO_2_ and residents of these neighborhoods had in turn a higher SBP, BMI, and CRP. In addition, proximity to waste/industrial facilities was a significant mediator between Carstairs and BMI: neighborhoods with greater deprivation had a higher proportion of residents living close to waste/industrial facilities, which was in turn linked to a higher BMI. PM_10_ was also a significant mediator between Carstairs and CRP (greater deprivation, higher PM_10_ levels, higher CRP).

Other significant mediation effects, albeit in unexpected directions, include: 1) PM_10_ partly mediating the association between Carstairs and SBP (neighborhoods with greater deprivation had higher levels of PM_10_, which were in turn linked to *lower* SBP); 2) CO partly mediating the association between Carstairs and BMI (greater deprivation, higher CO levels, *lower* BMI); and 3) NO_2_ partly mediating the association between Carstairs and CRP (greater deprivation, higher NO_2_ levels, *lower* CRP).

As for the moderation analyses, we found that the results for the whole sample were substantively similar to those for stratified samples (Additional file 1: Table S1). The finding that SO_2_ is a significant mediator between Carstairs and SBP, BMI and CRP seems to be particularly robust as it held for most of the stratified analyses. One exception was CRP: the positive mediating effects of SO_2_ and the negative mediating effects of NO_2_ found in the whole sample only remain for women when the sample was stratified by gender, with no significant mediating effects among men. Importantly, some of the unexpected mediation patterns observed for PM_10_ and SBP, CO and BMI, and NO_2_ and CRP also seem robust, holding true for most of the stratified analysis.

When stratifying by age, green space became a positive mediator between Carstairs and FEV_1_% in the 35–60 years age group, with exposure to greater neighborhood deprivation associated with reduced exposure to green space, which in turn was linked to lower FEV_1_%. Green space also emerged as a mediator between Carstairs and SBP for the younger age group (< 35 years) in an unexpected direction: greater deprivation was associated with reduced exposure to green space, which was in turned linked to lower SBP.

No clear patterns could be discerned between smoking groups, with the results overall mirroring those observed in the sample as a whole. The exception was for current smokers, for whom green space emerged as a significant mediator between Carstairs and CRP: greater neighborhood deprivation was associated with a reduced exposure to green space which was linked to higher CRP. In terms of the geographic stratifications, the unexpected mediating effects found for PM_10_ and SBP, CO and BMI, and NO_2_ and CRP remained for the urban sub-sample only, whereas no significant mediation effects were found for London, which is perhaps explained by the small sample sizes in these models (between *N* = 826 for CRP and *N* = 1418 for BMI).

## Discussion

This study contributes to the literature by empirically testing whether multiple physical environment characteristics explain the association between neighborhood socioeconomic deprivation and a set of objectively measured health outcomes. Using an MSEM framework, we were able to formally test for mediation looking at all the physical environment measures simultaneously, accounting for their existing interactions “in the real world” and decomposing the effects of each of the mediators as opposed to looking at the physical environment as a whole.

We found that neighborhood level of SO_2_ was a significant mediator between neighborhood socioeconomic deprivation and SBP, BMI and CRP. In Britain, SO_2_ emissions come primarily from combustion of fuel containing Sulphur, including coal and heavy oils processed in power stations and refineries [66]. Overall, the finding that exposure to SO_2_ is socially patterned [67] and that SO_2_ is detrimental to health, in particular blood pressure, BMI, and CRP, has support in the literature [20, 68, 69].

Surprisingly, none of the air pollutants (SO_2_ included) were mediators between neighborhood SEP and FEV_1_%, even though these pollutants were associated with both neighborhood deprivation and FEV_1_% in bivariate analysis, and they are absorbed into the body via the lungs, causing local inflammation *first* and then triggering a cascade of events linked with cardiovascular and/or systemic inflammation [30, 31]. We do not have an explanation for why SO_2_ level did not mediate the association between neighborhood deprivation and FEV_1_% but was a mediator for the other examined outcomes. It is plausible that the timing of events may have played a role; perhaps we are capturing the effects of chronic exposure to SO_2_ linked with living in deprived neighborhoods, which may in turn affect outcomes that take longer to develop, such as SBP and BMI, instead of the effects of an acute exposure, which would more likely affect the lungs. Also, maximum lung function capacity is determined in childhood, with studies relating air pollution exposure to lung function in adults finding very small effects [70]. It is possible, then, that early air pollutant exposure could mediate the association between childhood SEP and lung function, though this hypothesis cannot be tested with our current data.

While SO_2_ had a consistent association with most outcomes, except FEV_1_%, the other air pollutants had puzzling associations, such that PM_10_, CO and NO_2_ all negatively mediating deprivation-health associations for SBP, BMI and CRP, respectively. The main source of PM_10_, CO and NO_2_ in Britain is transportation [66]. These traffic-related pollutants were socially patterned in our sample, with a greater neighborhood deprivation associated with higher quantities of these pollutants, and more prevalent in urban areas. It is possible that areas with high levels of traffic (e.g. downtown areas) also have positive characteristics (e.g. increased access to jobs and services, increased walkability) which, in turn, provide health benefits that may confound the associations explored in the current analysis. This hypothesis seems to be supported by the fact that, when the sample was stratified by urbanity, the negative mediating effects of PM_10_ on SBP, CO on BMI, and NO_2_ on CRP remained in the urban sample but were not present among rural residents.

Another interesting finding related to air pollution was found for CRP when the sample was stratified by gender: for women, the mediating effects of SO_2_ (+) and NO_2_ (−) between Carstairs and CRP remained, with no significant mediating effects observed for men. Nazmi et al. [44] found that different neighborhood characteristics, including socioeconomic deprivation and air pollution, were linked to increased markers of inflammation among both men and women, with no evidence of gender interactions. However, other studies report that women’s health seems to be more affected by their physical neighborhood environment than men’s [42], either because of an increased exposure and/or an increased susceptibility.

We found no significant mediation effects for green space in the full sample. When we stratified by age, exposure to green space was a significant mediator between Carstairs and FEV_1_% for working-age adults (35–60 years), where greater neighborhood deprivation was associated with less green space and less green space was associated with lower FEV_1_%. Similar mediation effects were found for CRP among current smokers (greater deprivation, lower proportion of green space, lower CRP). Multiple studies have found health benefits for those exposed to green space, particularly an enhanced mental well-being [24, 71]. However, the relationships between access to green space, and its actual use, are known to be complex [72]; its presence does not guarantee use or benefits for all. Furthermore, the effects of green space on health seem to vary by sociodemographic characteristics. For example, Astell-Burt et al. [73] found that the positive impact of green space on mental health varied by age group and gender, with the beneficial effects of green space emerging among men in early to mid-adulthood. The interactions between age, gender and individuals’ SEP should be explored in the future when looking at green space as an explanatory factor between neighborhood poverty and health outcomes. In addition, the quality as well as the size and proximity of the green space available should be considered as some studies have found that this influences green space usage and, hence how positive green space exposure is for health [74, 75].

We found that proximity to waste and industrial facilities was a significant mediator between neighborhood socioeconomic deprivation and BMI, where higher deprivation was linked to closer proximity to waste/industrial facilities, which in turn was associated with increased BMI. When the sample was stratified by gender, this effect remained for women only., Since exposure to waste/industrial facilities was a mediator only for BMI, we could speculate the pathway at play may be psychosocial. Downey and Van Willigen [38] found that, among a representative sample in Illinois, USA, living closer to waste and industrial facilities was associated with increased perceptions of neighborhood disorder and decreased psychological well-being. Similarly, other research has highlighted the association between living proximally to waste and/or industrial facilities and high stress levels [39] and Matthews and Yang [40] found that living close to hazardous waste facilities strengthened the association between stress and health. The authors suggest their results follow the “hazardous waste syndrome,” in which proximity to waste facilities do not necessarily affect physical health but do so indirectly via stress [40]. In turn, both neighborhood disorder and stress have been linked to an increased obesity risk [76, 77].

### Strengths and limitations

The results obtained in this study highlight the challenges of investigating complex, interactive, and time-varying processes using cross-sectional designs. Some limitations inherent to most multilevel cross-sectional studies [78] include the issue of using artificial neighborhood boundaries, the inability to account for individual selection into neighborhoods, and the inability to establish the temporality of the association, since we could not account for people moving between neighborhoods and/or being exposed to more than one neighborhood. Moreover, capturing residence at one point in time may not represent individuals’ cumulative exposure to environmental hazards. Having longitudinal data on individuals’ residence linked to hazards and health at different time points would provide a clearer understanding of how these associations operate in a lifecourse perspective.

Exposure misclassification, of air pollutants in particular, is an important limitation which may explain some of our unexpected results; air pollutant levels vary on a very fine spatial scale and we treated all residents of a given CAS ward as having the same level of exposure. For other hazards, the scale at which they operate may vary; for example, the impact of green space may be much more immediate than that of a large industrial plant. Our results, therefore, may have been different if we had individual-level instead of area-level pollutant exposure or if we had focused on smaller geographies. The difference in data time frames (i.e. physical environment measures coming from 2001 to 2006 and biomarker data from 2010 to 2012) may also be problematic. However, we conducted sensitivity analysis with LSOAs, a smaller geography, and more recent data on air pollutants (from 2011), and the results of this analysis were qualitatively similar to those presented. While the specific indicators included in Carstairs may be less relevant today given social change, in general, there is a high level of correlation between areas that are deprived, or have poor physical environments, over time. It would have been informative to run analysis classifying neighborhoods as socioeconomically deprived vs. not deprived, based on appropriate cut-off points of the Carstairs index. This is methodologically challenging, however, as the MSEM framework does not lend itself to the introduction of categorical variables. Finally, even though UKHLS is based on a representative sample of UK residents, our sub-sample with biomarker data is not. We were unable to use sample weights given our multilevel design and the lack of sampling weights at the CAS ward level. Overall, those who participate in the nurse visit are older, richer and slightly healthier than those who do not [79]. Therefore, we anticipate that the results of this study may be an underestimation of the true associations.

This study does add unique new insights to the literature. It investigates several components of the physical environment simultaneously as possible mediators in the association between neighborhood socioeconomic deprivation and health, focusing on a large sample of British residents. Given the large sample size, we were able to investigate whether the associations held true for key subgroups of the population, although further investigations might explore interactions in these moderating effects. Both our physical environment measures as well as our health variables were objectively measured, and the included biomarkers represent different systems within the body (i.e. respiratory, cardiovascular, inflammatory systems). Further, by using a more robust statistical method (MSEM framework) we were able to account for possible interactions between the physical environment measures in the model.

## Conclusion

Living in neighborhoods with poor physical environment characteristics was associated with worse health outcomes, but only some of these characteristics mediated the association between neighborhood socioeconomic deprivation and health. However, as this is an exploratory study, future research should try to replicate our findings in different populations. If these findings hold, changing the physical environment may improve population’s health but may not decrease health inequalities. The one exception was exposure to SO_2_, which was a clear and robust mediator in the association between neighborhood deprivation and SBP, BMI, and CRP. In Britain, SO_2_ comes primarily from coal burning and petroleum production. Focusing public resources on alternative green energy production, therefore, should not only be a priority for combating climate change, but also part of the agenda to decrease social inequalities in health.

## Additional file

Additional file 1: **Table S1.** Qualitative summary of significant mediation effects in stratified samples by age, gender, smoking status, urbanity, and region. Results from complete case and stratified analyses of the data as sensitivity tests for main findings. (DOCX 20 kb)

## Abbreviations

BHPS: British Household Panel Study
BMI: Body mass index
CAS: Census area statistic ward
CO: Carbon monoxide
CRP: C-reactive protein
ERS-GLI: European Respiratory Society Global Lung Function Initiative
FEV_1_: Forced expiratory volume in 1 s
FEV_1_%: Percent predicted FEV_1_
MEDIx: Multiple Environmental Deprivation Index
MLR: Maximum likelihood with robust standard errors
MSEM: Multilevel structural equation modeling
NO_2_: Nitrogen dioxide
PM_10_: Particulate matter
SBP: Systolic blood pressure
SEP: Socioeconomic position
SO_2_: Sulphur dioxide
UHHLS: The UK Household Longitudinal Survey
