# Supplementary material for: Neighborhood deprivation and biomarkers of health in Britain: the mediating role of the physical environment
Source: BMC Public Health. 2018 Jun 27;18:801. doi: 10.1186/s12889-018-5667-3 (PMC6020450; doi:10.1186/s12889-018-5667-3)
Supplement: Supplementary file 1 — Table S1. Qualitative summary of significant mediation effects in stratified samples by age, gender, smoking status, urbanity, and region. Results from complete case and stratified analyses of the data as sensitivity tests for main findings. (DOCX 20 kb) [file 12889_2018_5667_MOESM1_ESM.docx]

**Additional file 1: Table S1: Qualitative summary of significant mediation effects in stratified samples by age, gender, smoking status, urbanity, and region**

|  | **FEV_1_%** | **SBP** | **BMI** | **CRP** |
| --- | --- | --- | --- | --- |
| **By age groups**  <35 years        35-60 years      >60 years | None  Green space (GS, -)  ↑deprivation ↓GS  ↑GS ↑FEV_1_%  None | SO_2_ (+)  ↑deprivation ↑SO_2_  ↑SO_2_ ↑SBP  Green space (GS, -)  ↑deprivation ↓GS  ↑GS ↑SBP  SO_2_ (+)  ↑deprivation ↑SO_2_  ↑SO_2_ ↑SBP  PM_10_ (-)  ↑deprivation ↑PM_10_  ↑PM_10_ ↓SBP  SO_2_ (+)  ↑deprivation ↑SO_2_  ↑SO_2_ ↑SBP  PM_10_ (-)  ↑deprivation ↑PM_10_  ↑PM_10_ ↓SBP | SO_2_ (+)  ↑deprivation ↑SO_2_  ↑SO_2_ ↑BMI  SO_2_ (+)  ↑deprivation ↑SO_2_  ↑SO_2_ ↑BMI  CO (-)  ↑deprivation ↑CO  ↑CO ↓BMI  CO (-)  ↑deprivation ↑CO  ↑CO ↓BMI | None  SO_2_ (+)  ↑deprivation ↑SO_2_  ↑SO_2_ ↑CRP  PM_10_ (+)  ↑deprivation ↑PM_10_  ↑PM_10_ ↑CRP  NO_2_ (-)  ↑deprivation ↑NO_2_  ↑NO_2_ ↓CRP  CO (+)  ↑deprivation ↑CO  ↑CO ↑CRP |
| **By gender**  Women        Men | None  None | SO_2_ (+)  ↑deprivation ↑SO_2_  ↑SO_2_ ↑SBP  PM_10_ (-)  ↑deprivation ↑PM_10_  ↑PM_10_ ↓SBP  SO_2_ (+)  ↑deprivation ↑SO_2_  ↑SO_2_ ↑SBP  PM_10_ (-)  ↑deprivation ↑PM_10_  ↑PM_10_ ↓SBP | SO_2_ (+)  ↑deprivation ↑SO_2_  ↑SO_2_ ↑BMI  Industrial facilities (IF, +)  ↑deprivation ↑IF  ↑IF ↑BMI  SO_2_ (+)  ↑deprivation ↑SO_2_  ↑SO_2_ ↑BMI  CO (-)  ↑deprivation ↑CO  ↑CO ↓BMI | SO_2_ (+)  ↑deprivation ↑SO_2_  ↑SO_2_ ↑CRP  NO_2_ (-)  ↑deprivation ↑NO_2_  ↑NO_2_ ↓CRP  CO (+)  ↑deprivation ↑CO  ↑CO ↑CRP  None |
| **By smoking status**  Never smoker      Former smoker          Current smoker | SO_2_ (+)  ↑deprivation ↑SO_2_  ↑SO_2_ ↑FEV_1_%  None  None | SO_2_ (+)  ↑deprivation ↑SO_2_  ↑SO_2_ ↑SBP  SO_2_ (+)  ↑deprivation ↑SO_2_  ↑SO_2_ ↑SBP  PM_10_ (-)  ↑deprivation ↑PM_10_  ↑PM_10_ ↓SBP  SO_2_ (+)  ↑deprivation ↑SO_2_  ↑SO_2_ ↑SBP  PM_10_ (-)  ↑deprivation ↑PM_10_  ↑PM_10_ ↓SBP | SO_2_ (+)  ↑deprivation ↑SO_2_  ↑SO_2_ ↑BMI  CO (-)  ↑deprivation ↑CO  ↑CO ↓BMI  SO_2_ (+)  ↑deprivation ↑SO_2_  ↑SO_2_ ↑BMI  CO (-)  ↑deprivation ↑CO  ↑CO ↓BMI  SO_2_ (+)  ↑deprivation ↑SO_2_  ↑SO_2_ ↑BMI | None  SO_2_ (+)  ↑deprivation ↑SO_2_  ↑SO_2_ ↑CRP  NO_2_ (-)  ↑deprivation ↑NO_2_  ↑NO_2_ ↓CRP  CO (+)  ↑deprivation ↑CO  ↑CO ↑CRP  PM_10_ (+)  ↑deprivation ↑PM_10_  ↑PM_10_ ↑CRP  NO_2_ (-)  ↑deprivation ↑NO_2_  ↑NO_2_ ↓CRP  Green space (+)  ↑deprivation ↓GS  ↑GS ↓CRP |
| **By urbanity**  Urban        Rural | PM_10_ (-)  ↑deprivation ↑PM_10_  ↑PM_10_ ↓FEV_1_%  PM_10_ (-)  ↑deprivation ↑PM_10_  ↑PM_10_ ↓FEV_1_% | SO_2_ (+)  ↑deprivation ↑SO_2_  ↑SO_2_ ↑SBP  PM_10_ (-)  ↑deprivation ↑PM_10_  ↑PM_10_ ↓SBP  PM_10_ (+)  ↑deprivation ↑PM_10_  ↑PM_10_ ↑SBP | SO_2_ (+)  ↑deprivation ↑SO_2_  ↑SO_2_ ↑BMI  CO (-)  ↑deprivation ↑CO  ↑CO ↓BMI  None | SO_2_ (+)  ↑deprivation ↑SO_2_  ↑SO_2_ ↑CRP  PM_10_ (+)  ↑deprivation ↑PM_10_  ↑PM_10_ ↑CRP  NO_2_ (-)  ↑deprivation ↑NO_2_  ↑NO_2_ ↓CRP  None |
| **By region**  London    The rest of the UK | None  None | None  SO_2_ (+)  ↑deprivation ↑SO_2_  ↑SO_2_ ↑SBP  PM_10_ (-)  ↑deprivation ↑PM_10_  ↑PM_10_ ↓SBP | NO_2_ (-)  ↑deprivation ↑NO_2_  ↑NO_2_ ↓BMI  SO_2_ (+)  ↑deprivation ↑SO_2_  ↑SO_2_ ↑BMI | None  PM_10_ (+)  ↑deprivation ↑PM_10_  ↑PM_10_ ↑CRP  NO_2_ (-)  ↑deprivation ↑NO_2_  ↑NO_2_ ↓CRP  CO (+)  ↑deprivation ↑CO  ↑CO ↑CRP |
